# Stress, fear, and anxiety among construction workers: a systematic review

**DOI:** 10.3389/fpubh.2023.1226914

**Published:** 2023-07-13

**Authors:** Carlos Gómez-Salgado, Juan Carlos Camacho-Vega, Juan Gómez-Salgado, Juan Jesús García-Iglesias, Javier Fagundo-Rivera, Regina Allande-Cussó, Jorge Martín-Pereira, Carlos Ruiz-Frutos

**Affiliations:** ^1^School of Doctorate, University of Huelva, Huelva, Spain; ^2^Department of Building Construction II, Higher Technical School of Building Engineering, University of Seville, Andalucia, Spain; ^3^Department of Sociology, Social Work and Public Health, Faculty of Labour Sciences, University of Huelva, Huelva, Spain; ^4^Escuela de Posgrado, Universidad de Especialidades Espíritu Santo, Guayaquil, Ecuador; ^5^Red Cross Nursing University Centre, University of Seville, Seville, Spain; ^6^Department of Nursing, University of Seville, Seville, Spain

**Keywords:** anxiety, stress, fear, mental health, construction workers, construction industry, work conditions

## Abstract

**Objectives:**

The aim of this review was to assess the possible risk factors arising from working conditions, that could have an impact on the stress, fear, and anxiety of construction workers.

**Methods:**

A systematic review was conducted following the PRISMA format in the Pubmed, Cochrane, Web of Science, Scopus, and PsycInfo electronic databases on February 3, 2023, using the following key words: anxiety, stress, fear, and construction workers. Methodological quality was assessed using the critical appraisal tools of the Joanna Briggs Institute.

**Results:**

A total of 35 studies were included. The results showed a number of conditioning factors for stress, anxiety, and fear among construction workers such as age, inappropriate safety equipment, safety culture, high workload and long working hours, physical pain, low social support from direct supervisor or co-workers, lack of organizational justice and lack of reward, financial situation, maladaptive coping strategies, and characteristics of the pandemic.

**Conclusions:**

There are a number of risk factors related to working conditions, organizations, and individuals that can affect the levels of stress, anxiety, and fear among construction workers, such as age, work hardship, safety culture and, especially, the long hours that construction professionals work. This may lead to an increase in the number of occupational accidents and higher associated fatality rates.

**Systematic review registration:**

https://www.crd.york.ac.uk/prospero/display_record.php?ID=CRD42022367724, identifier: CRD42022367724.

## Introduction

The construction sector is currently one of the main productive sectors and economic engines in most countries (1). It is in constant change, evolving as techniques improve and new technological advances appear. Despite this, it is one of the sectors with the highest mortality rates worldwide (2, 3), as it involves complex and hazardous activities such as construction itself, damming, road construction, engineering activities, demolition of all types of structures, rehabilitation and maintenance of structures, among others (4). These activities may involve a certain degree of danger if appropriate protective measures are not taken as they involve working at heights and with electrical hazards, exposure to high temperatures, excessive noise, chemical handling and dusty environment, carrying heavy equipment, handling heavy loads, and using heavy machinery (5). These circumstances or factors, which are not exceptional but common in their activity, make these workers carry out their work in harsh conditions that involve constant efforts and in environmental conditions that make their work difficult.

The tasks that construction workers perform may be found to be unsatisfactory for them due to the concurrence of factors related to the work itself, individual characteristics, lifestyle and concomitant health problems, and/or problems related to the professional performance itself (6, 7). In fact, these are considered to be high stress environments (8) and in which mental health problems appear to be growing (9). Stress among construction workers can lead to other problems on a physical level such as musculoskeletal disorders, on a mental level such as anxiety, and it can reduce productivity through absenteeism and presenteeism (10) and lead to errors that may endanger the safety and health of workers and co-workers (7).

Stress can be considered as the body's response to frequent and/or continuous mismatches between an individual's demands and the individual's ability to cope with them (11). This has direct physiological effects on the person and also affects health when our health maintenance behaviors are altered (12). The transactional model is one of the most prestigious models of the psychosocial stress process. Lazarus calls it “transactional” because it states that stress originates neither in the person nor in the environment, but in the interaction between the two (13). On the other hand, the Job Demands-Resources (JD-R) model developed by Bakker and Demerouti (14) provides an insight into how a mismatch between work demands and resources can lead to mental health problems such as stress, anxiety, and fear. But mental diseases are not only caused by factors intrinsic to the construction site. Other studies have shown that the socio-cultural environment in which the worker lives is a key factor for the development of mental illnesses, many of which are associated with the consumption of alcohol and other substances (15).

It is well known that construction projects often have very tight deadlines. This means that the teams of people who carry out the work, whether they are craftsmen, site teams, supervisors, or technical staff, are under a lot of pressure from their companies. In addition to stress, this can cause anxiety in the worker. Anxiety, according to Spielberger (16), can be divided into state anxiety and trait anxiety. While the former is a temporary and situational state of emotion in response to a threat, the latter is part of the personality of each individual. Although there are several studies that have assessed the levels of anxiety, stress, and fear in construction workers, each of them addresses a number of specific factors that may increase the levels of these three variables, but it is uncertain which were analyzed as risk factors for each variable (anxiety, stress, and fear) and in each study. In a sector with a high number of occupational accidents, knowing the factors could be a useful tool for establishing possible effective protective and preventive measures for these workers, and could help future researchers to consider and prioritize some risk factors over others. Therefore, the aim of the study was to assess the factors influencing stress, fear, and anxiety among construction workers.

## Methods

### Study design

In order to assess the risk factors related to levels of anxiety, stress, and fear among construction workers in the construction industry, a systematic review was conducted following the guidelines of the PRISMA statement (Preferred Reporting Items for Systematic reviews and Meta-Analyses) (17). The protocol followed is listed in the International Prospective Register of Systematic Reviews (PROSPERO) with code CRD42022367724.

### Databases and search strategy

The search was carried out in the Pubmed, Cochrane Library, Web of Science Scopus, and PsycInfo electronic databases on the basis of the keywords that the research question yielded following the PECO strategy (Table 1).

**Table 1:** PECO format: keywords (Spain, 2023).

**Population**	**Construction workers**
Effect	Alteration of stress, fear, and anxiety levels
Comparator	Identify influential risk factors
Outcomes	Level of burnout, stress, anxiety, and fear, number of cases of people with depression/stress/anxiety/fear, substance use, insomnia, physical manifestations of stress, comparison according to type of profession/sex/country, possible risks for the occurrence of accidents at work, coping measures, how working and/or psychosocial conditions influence, and health-work relationship and vice versa.
Research question	What factors influence stress, fear, and anxiety of workers in the construction sector?

Based on these keywords, the Medical Subject Headings (MeSH) thesaurus was consulted, yielding the descriptors Anxiety, Psychological Stress, Fear, and Construction industry. In order to enlarge the scope of the search, synonymous terms were used to complete the search based on the Medical Subject Headings (MeSH) thesaurus (Table 2), linked by the Boolean operators AND and OR.

**Table 2:** Terms used in the search (Spain, 2023).

**MeSH**	**Meaning**	**Terms**
Anxiety	Feelings or emotions of dread, apprehension, and impending disaster but not disabling as with anxiety disorders	Anxiety
Psychological stress	Stress wherein emotional factors predominate	Stress
Fear	The affective response to an actual current external danger which subsides with the elimination of the threatening condition	Fear
Construction industry	The aggregate business enterprise of building	Construction workers OR building industry

Table 3 shows the search strategy used, carried out on February 3, 2023, for each of the above databases during the search process for articles published in the last 10 years.

**Table 3:** Search strategy (Spain, 2023).

**Database**	**Search strategy**	**Results**
Pubmed	(construction workers [Title/Abstract] OR building industry [Title/Abstract]) AND (anxiety [Title/Abstract] OR stress [Title/Abstract] OR fear [Title/Abstract]) Filters: from 2012–2023	86
Cochrane	(anxiety):ti,ab,kw OR (stress):ti,ab,kw OR (fear):ti,ab,kw AND (“construction worker”):ti,ab,kw OR (building industry):ti,ab,kw “with Cochrane Library publication date Between Jan 2012 and Jan 2023 (Word variations have been searched)	120
Web Of Science	anxiety OR stress OR fear (Topic) AND (“construction workers”) OR (“building industry”) (Topic) and 2023 or 2022 or 2021 or 2020 or 2019 or 2018 or 2017 or 2016 or 2015 or 2014 or 2013 or 2012 (Publication Years) and Review Article (Exclude—Document Types)	469
Scopus	(TITLE-ABS-KEY (anxiety OR stress OR fear) AND TITLE-ABS-KEY (“construction workers” OR “building industry”)) AND (LIMIT-TO (PUBYEAR, 2023) OR LIMIT-TO (PUBYEAR, 2022) OR LIMIT-TO (PUBYEAR, 2021) OR LIMIT-TO (PUBYEAR, 2020) OR LIMIT-TO (PUBYEAR, 2019) OR LIMIT-TO (PUBYEAR, 2018) OR LIMIT-TO (PUBYEAR, 2017) OR LIMIT-TO (PUBYEAR, 2016) OR LIMIT-TO (PUBYEAR, 2015) OR LIMIT-TO (PUBYEAR, 2014) OR LIMIT-TO (PUBYEAR, 2013) OR LIMIT-TO (PUBYEAR, 2012)) AND (EXCLUDE (DOCTYPE, “cp”) OR EXCLUDE (DOCTYPE, “re”) OR EXCLUDE (DOCTYPE, “ch”) OR EXCLUDE (DOCTYPE, “no”) OR EXCLUDE (DOCTYPE, “ed”) OR EXCLUDE (DOCTYPE, “cr”) OR EXCLUDE (DOCTYPE, “bk”) OR EXCLUDE (DOCTYPE, “le”))	437
PsycInfo	tiab(anxiety OR stress OR fear) AND tiab((“construction worker” OR “construction workers”) OR “building industry”) Date: after 2012	38
**Date of search:** February 3, 2023	**Total**	1,150

### Selection criteria

The following inclusion criteria were used for the selection of articles: (1) original articles published in English, Spanish, French, and Portuguese; (2) typology: original articles, meta-analysis, short communications, and case reports; (3) articles published in the last 10 years; and (4) articles measuring any of the following values and/or effects: level of stress, anxiety and fear, number of cases of people with stress/anxiety/fear, substance use, insomnia, physical manifestations of psychological stress, comparison according to type of profession/sex/country, possible risks for the materialization of accidents at work, coping measures, how work and/or psychosocial conditions influence, and health-work relationship and vice versa. Similarly, the exclusion criteria were: (1) studies in a language other than English, Spanish, French, and Portuguese; (2) typology: opinion articles, editorials, and letters to the editor; (3) studies of low scientific-technical quality after applying the quality assessment tool; and (4) articles that did not answer the research question and were not related to the objective of the review.

### Data collection and extraction

For this search, a pre-established protocol was initially followed for the search and revision strategy in order to minimize the risk of bias in the selection and subsequent publication. This strategy was similar in the different databases by using the aforementioned descriptors and related keywords through the Boolean operators AND and OR. In the drafting of this work, two researchers independently carried out the bibliographic searches. As a secondary strategy, a search was carried out based on the use of references and names of the authors cited in the different records selected (reverse or snowball search) with the intention of verifying the existence of works not found in the primary search. For the screening and selection of articles, duplicate studies were eliminated and those articles that could be included were selected after reading the abstract and title according to the previously established criteria. After this initial screening, the same authors analyzed the full articles and selected those studies potentially suitable for inclusion in the review. This selection was made by consensus between the two researchers and any discrepancies that may have arisen were resolved by a third author.

### Methodological quality assessment

Two reviewers independently determined the methodological quality of the selected studies using the critical appraisal tools of the Joanna Briggs Institute (JBI) at the University of Adelaide. These tools allow the assessment of the methodological quality of a study and the extent to which a study has excluded or minimized the possibility of bias in its design, conduct, and/or analysis. The versions for analytical cross-sectional studies (8 items) (Supplementary Table S1), for qualitative research (10 items) (Supplementary Table S2), and for randomized controlled trials (12 items) (Supplementary Table S3) were used, setting the cut-off point at 6–9, respectively, for inclusion in this review. The included studies were assessed and the mean scores were obtained.

## Results

**Figure 1:**
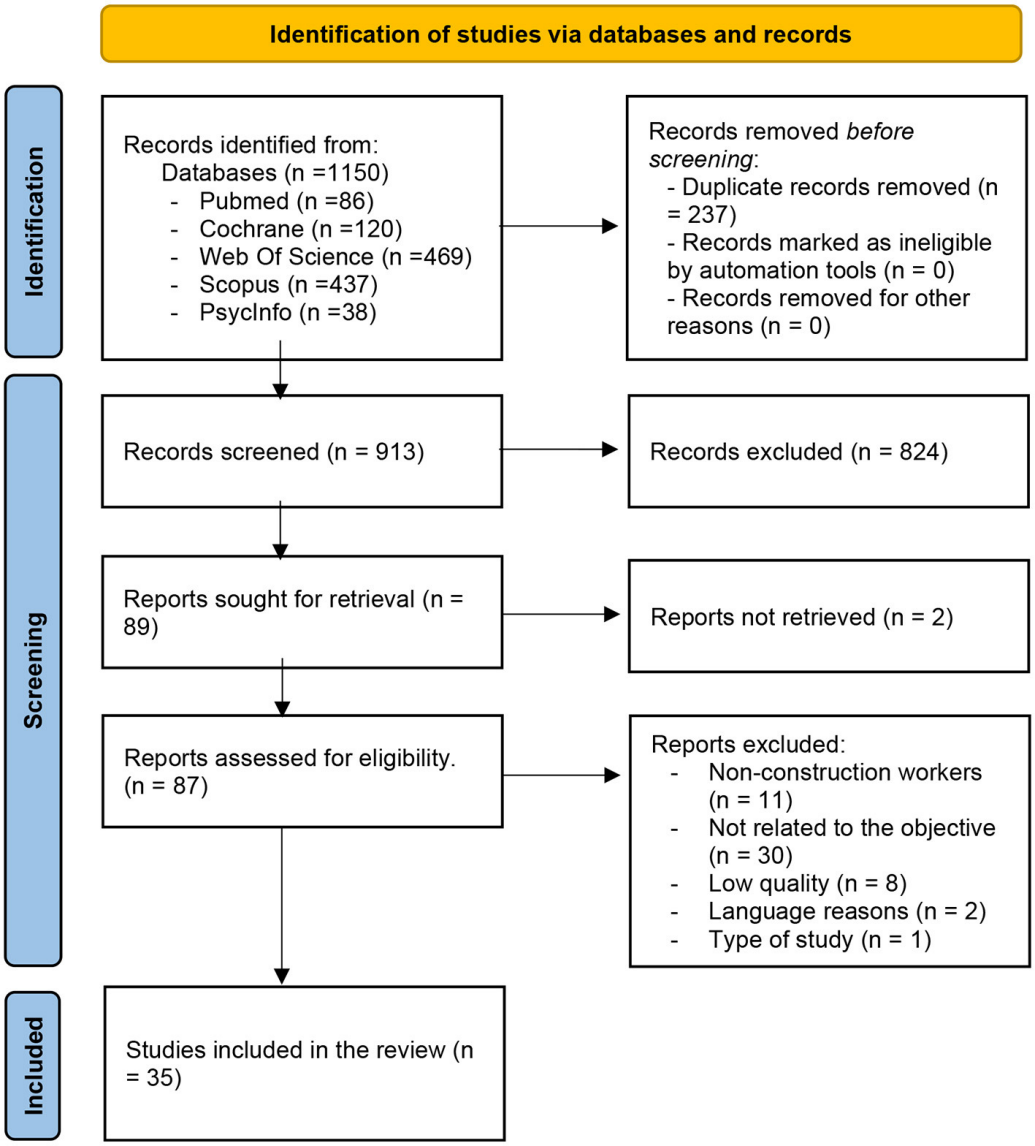
Search results (PRISMA Flowchart) (Spain, 2023).

A total of 35 studies were selected. The initial search strategies identified a total of 1,150 references, which were then screened according to the topic of this review. Twenty-six of the 35 studies were analytical cross-sectional studies, 2 carried out qualitative research, 6 were mixed methods, and 1 was a quasi-experimental study (Figure 1).

Table 4 shows the characteristics of each of the 35 studies included in this review. These were classified by author and year of publication, country, design and objective, participants, instrument, and main results. In addition, the results of the JBI critical appraisal tool were added.

**Table 4:** Characteristics of the studies included in the systematic review (Spain, 2023).

**References**	**Context**	**Study objective**	**Type of study**	**Participants**	**Instruments**	**Main findings**	**Quality of the studies**
Leung et al. (18)	China	To identify the impact of various organizational stressors and stress on CWs safety behaviors and injury incidents.	Quantitative cross-sectional study	CWs (*n* = 395)	- Organizational stressor scale - Emotional stress scale - Physical stress scale - Safety behavior scale	Injury incidents were minimized by safety behaviors but escalated by a lack of goal setting; safety behaviors were maximized by moderate levels of emotional stress and increased in line with physical stress and inappropriate safety equipment; emotional stress was positively predicted by the provision of training and inappropriate safety equipment; and physical stress was predicted only by inappropriate safety equipment.	8/8
Boschman et al. (19)	Netherlands	To assess the magnitude of psychosocial work characteristics; the prevalence of self-reported mental health effects; and the psychosocial factors that are associated with mental health.	Quantitative cross-sectional study	Bricklayers (*n* = 750) Supervisors (*n* = 750)	- QWWE - FDW - Distress screener - IES - 4DSQ	10.9% of bricklayers had symptoms of PTSD; 4.7% were distressed; and 17.6% had symptoms of depression. Among the supervisors, 6.9% had symptoms of PTSD; 6.8% were distressed; and 19.6% had symptoms of depression. Among bricklayers, high work speed and quantity were associated with symptoms of depression (OR 4.1; 1.2–14.3). For supervisors, high work speed and quantity (OR 2.8, 1.0–7.7), low participation in decision making (OR 5.5; 1.7–17.9) low social support of the direct supervisor (OR 7.5; 1.9–30.0) were associated with symptoms of depression. Among supervisors, high workload (OR 5.6; 1.1–28.1) was associated with distress.	6/8
Jacobsen et al. (20)	USA	To investigate how mental distress was associated with pain and injuries in a convenience sample of CWs.	Mixed methods approach	CWs (*n* = 172) Clinical interview (*n* = 10)	- Hopkins symptom checklist-25 - K6	The prevalence of substantial mental distress was 16% in CWs. This was supported by follow-up clinical interviews where 9 of 10 workers fulfilled the criteria for a mental disorder. Substantial mental distress was associated with both injury rate and self-reported pain.	8/8
Hammer et al. (21)	USA	To assess a TWH intervention, the Safety and Health Improvement Program (SHIP), designed to address work-family stress and safety risk factors.	Randomized controlled trials	CWs (*n* = 198)	- FSSB and SBS - TEP process - Safety behaviors - SF-12 - Blood pressure	No significant differences across intervention conditions were observed at baseline for blood pressure (*B* = 0.31; *p* = 0.84), SF-12 physical health composite scores (*B* = 1.23; *p* = 0.18), safety compliance (*B* = −0.08; *p* = 0.42), and safety participation (*B* = −0.29; *p* = 0.99). There are negative correlations between age and SF-12 physical health composite scores, a negative correlation between taking blood pressure medication and SF-12 physical health composite scores, and a positive correlation between taking blood pressure medication and age.	9/12
Seo et al. (22)	Korea	To develop a research model based on: individual factors, job stress, self-perceived fatigue and factors affecting safety behavior.	Quantitative cross-sectional study	CWs (*n* = 415)	- Individual factors - Job stress - Self-perceived fatigue - Safety behavior	First, personal characteristics had a partial effect on job stress and a direct effect on safety culture. Second, personal characteristics and job stress had a direct effect on self-perceived fatigue. Third, personal characteristics and safety culture had a direct effect on safety climate, and personal characteristics also had an indirect effect.	7/8
Leung et al. (23)	China	To investigate the relationships between job stressors, stress, safety behavior, and accidents.	Quantitative cross-sectional study	CWs (*n* = 166)	- Job stressors - Stress - Safety behavior	Physical stress is predicted by job certainty, co-worker support, and safety equipment, while psychological stress is predicted by both supervisor support and job certainty. Supervisor support and physical stress predict safety behavior; and the risk of accidents can be reduced by safety behavior, whereas a high level of job control increases it.	6/8
Chen et al. (24)	Canada	To examine the role of safety climate and individual resilience in safety performance and job stress in the Canadian construction industry.	Quantitative cross-sectional study	CWs (*n* = 837)	- Demographics - Attitude statements - Incident reporting	The results show that safety climate not only affected construction workers' safety performance but also indirectly affected their psychological stress. In addition, it was found that individual resilience had a direct negative impact on psychological stress but had no impact on physical safety outcomes, safety climate explained 7 and 6% variance of physical symptoms and unsafe events, respectively. IR explained 3% variance of stress symptoms, physical symptoms contributed 17%, and unsafe events contributed 9%.	7/8
Lim et al. (25)	Korea	To understand the level of psychological conditions of construction field-workers using four categories: (1) stress (occupational stress and stress-coping style), (2) personal temperament, (3) emotional disturbance (depression and trait anxiety), and (4) drinking habits.	Quantitative cross-sectional study	CWs (*n* = 430)	- KOSS-SF - TCI-RS - CES-D - STAI - AUDIT	CWs suffer from a high level of stress and showed high inclination for problem-focused coping: impulsive, cautious, and dependent on other people. Two out of five construction workers suffer from depression and experience trait anxiety. More seriously, three out of five workers show alcohol-use problems that require clinical attention. This study also revealed the particular psychological problems that occur under different working conditions.	8/8
Bowers et al. (26)	Australia	To assess the prevalence and correlates of psychological distress in a sample of remote mining and construction workers in Australia.	Quantitative cross-sectional study	Mining and CWs (*n* = 1,124)	- Self-reported overall mental health status - K10	The most frequently reported stressors were missing special events (86%), relationship problems with partners (68%), financial stress (62%), shift rosters (62%), and social isolation (60%). High psychological distress was significantly more likely in workers aged 25–34 years and workers on a 2 weeks on/1 week off roster. Workers who were very or extremely stressed by their assigned tasks or job, their current relationship, or their financial situation were significantly more likely to have high/very high K10 scores than those not stressed by these factors. Workers who reported stress related to stigmatization of mental health problems were at the greatest risk of high/very high psychological distress.	8/8
Chakraborty et al. (27)	India	To evaluate the occupational stress and other factors in the prevalence of musculoskeletal disorders and their impact on the quality of life of these workers.	Quantitative cross-sectional study	CWs (*n* = 268)	- OSI - NMQ - WHOQoL-BREF	CWs worked long hours and were burdened with stress (10.43 h/day and 68.14 h/week), with a score of 76.76. 39.92% of workers worked for more than 12 h per day. Most of the workers reported musculoskeletal pain in the body parts that were mostly used during the tasks performed (80% percentage of respondents experienced some form of MSDs in the past 12 months). These workers scored poor in all the domains of the quality of life.	8/8
Langdon and Sawang (28)	Australia	To determine (1) the daily primary stressors in the construction workplace as identified by CWs; and (2) the relationships between the strain effect of psychological distress and the countermeasures and coping mechanisms used by construction workers (depression, anxiety, and stress).	Mixed methods approach	CWs in different occupations (*n* = 18 qualitative study) (*n* = 91 quantitative study).	- Q methodology - DASS-21 - BCI - Sobel test	Long working hours/weeks, lack of personal and family time, increases in the cost of living, and fears about job security all act as powerful stressors for workers and potentially affect psychological outcomes. The maladaptive coping strategies together (self-distraction, denial, substance use, behavioral disengagement, venting, and self-blame) explained 78.9% of the variance in depression, 63% in anxiety, and 60.3% in stress. Increased substance use, although associated with lower levels of anxiety, may only be a short-term coping mechanism.	6/8
Liang et al. (29)	China	To explore the participants' true opinions and feelings about their coping behaviors, stress, and performance.	Qualitative study	skilled CWs (*n* = 8); general CWs (*n* = 6); supervisors (*n* = 10)	Focus group (interview).	The study revealed that CWs experienced more than 10 types of both emotional and physical stress symptoms. In addition to physical stress symptoms, CWs simultaneously experience five emotional stress symptoms, including anxiety, being angry, tension, listlessness, and worrying. Stress was also found to be one reason why CWs leave a company. The participants mentioned that the various stress symptoms cause CWs to engage in unsafe behaviors, leading to high accident rates.	8/10
Maqsoom et al. (30)	Pakistan	To examine the intrinsic (top management, career development, social support, motivation, and work stress) psychosocial stressors that influence the productivity of Pakistani construction contracting firms workers having varied ages and industry experiences.	Quantitative cross-sectional study	CWs (*n* = 163)	- Worker productivity and project performance - Psychosocial stressors	Employees of varied ages did not concur over several top management, career development, social support, motivation and work stress related psychosocial stressors, whereas employees of varied industrial experience were in disagreement over some work stress related psychosocial stressors. Job-related stress is reduced considerably in the presence of co-workers' support (mean rank = 97.27 for younger workers and 72.13 for older workers with a significance of 0.000).	7/8
Wang et al. (31)	China	To examine the predictive powers of safety-related stress and psychological capital on safety behavior, and the moderating role of psychological capital on the safety-related stress–behavior relationship	Quantitative cross-sectional study	CWs (*n* = 359)	- Safety-related stress - PsyCap-24 scale - Safety behavior	High safety-related stress would impair safety behavior in terms of safety participation but not safety compliance. Psychological capital's positive influence on safety compliance was stronger than that on safety participation. Furthermore, psychological capital moderated the relationship between safety-related stress and safety participation. For their sub-dimensions, it was found that (1) three selected safety-related stressors had negative influences on safety participation, while only safety role ambiguity had an effect on safety compliance; (2) four sub-dimensions of psychological capital had stronger influences on safety compliance than those on safety participation; (3) general psychological capital moderated the three safety-related stressors' effects on safety participation; and (4) four sub-dimensions of psychological capital moderated the effect of general safety-related stress on safety participation.	8/8
Widajati (32)	Indonesia	To develop a problem focus coping model mechanism against environmental stressors to prevent unsafe work action in steel CWs at production line.	Quantitative cross-sectional study	Steel CWs (*n* = 150)	Stressors in the work environment	Mild stress levels were experienced by as many as 80 workers, stress by as many as 65 workers, and severe stress by much as 5 workers. The effects of environmental stressors on working with unsafe behavior are very significant (*P* = 0.003).	6/8
Yaldiz et al. (33)	USA	To examine the age-related differences in the usefulness of job resources in relation to employee stress, an important wellbeing outcome.	Quantitative cross-sectional study	CWs (*n* = 348)	- JCQ - Relationship with supervisor - Procedural fairness (organization) - Employee stress	Age was positively related to perceived stress. Job tenure was positively associated with both perceived stress and employee age. Skill discretion was not related to perceived stress in younger workers while it was negatively related to perceived stress in older workers. There is a negative relationship between LMX and perceived stress in older workers. However, for younger workers, LMX was not related to perceived stress. A negative relationship between procedural fairness and perceived stress was confirmed in older workers, unlike in younger workers.	6/8
Hampton et al. (34)	United Kingdom	To investigate 3 different aspects of stress: (1) the stress factors; (2) the consequences of stress and their impacts on CWs; (3) the tools and measures to cope with stress.	Qualitative study	CWs (construction sites and offices) (*n* = 39)	Ethnographic enquiry: - Observation - Field notes - Interviews	“Ambiguity” represents an important variable of stress which creates a challenging environment characterized by limited time, poor communication and sometimes limited resources and facilities. “Teamwork” is another important factor of stress. This principle is even more important for construction workers because they work close to each other physically and temporally. The level of stress experienced by workers is strictly connected with their level of engagement and commitment in handling responsibilities: the more engaged they are in their activities, the more likely the possibility of their experiencing a high level of stress.	9/10
He et al. (35)	China	To test the relationship between sub-dimensions of PsyCap (self-efficacy, hope, resilience, and optimism) and safety behaviors (safety compliance, safety participation). To explore the mediating role of communication competence.	Quantitative cross-sectional study	CWs (*n* = 655)	PCQ.	The self-efficacy dimension of PsyCap positively affected safety compliance and safety participation, while the resilience dimension positively impacted safety participation. The hope dimension was not directly related to safety behaviors, while the optimism dimension negatively associated with safety participation. Communication competence mediated the relationships between the hope and optimism dimensions of PsyCap and safety participation.	8/8
Hussen et al. (36)	Ethiopia	To determine the prevalence and associated factors of occupational injury among Genale Dawa hydropower dam CWs.	Quantitative cross-sectional study	Hydropower Dam CWs (*n* = 405)	- Variables: smoking cigarettes, drinking alcohol chewing khat, sleeping problem, job satisfaction, job stress, and using PPE.	Study participants with job stress were 3.47 times more likely to be injured when compared to subjects who had not encountered job stress [AOR: 3.47, 95% CI (1.90, 6.35)].	6/8
Jung et al. (37)	Korea	To investigate how the work environment and psychological state influence CWs' perceptions and safety behaviors.	Quantitative cross-sectional study	CWs (*n* = 399)	- JCQ - OSI - JSQ - CES-D - STAI	Depression was mediated by safety motivation (β = −0.263, *p* = 0.000) and trait anxiety mediated by safety knowledge (β = −0.168, *p* = 0.000) when they influenced safety compliance and participatory behavior. It was partially adopted that the psychological condition mediates the working environment's impact on safety behavior. Job demand (β = 0.180, *p* = 0.003) and the lack of organizational justice (β = 0.204, *p* = 0.003) indirectly affected safety behavior through depression (H6a), and the lack of reward (β = 0.364, *p* = 0.000) was mediated by anxiety (H6b).	8/8
Roche et al. (38)	Australia	To investigate the patterns, prevalence and predictors of risky drinking among CWs	Quantitative cross-sectional study	CWs (*n* = 511)	- AUDIT-C - K10 - Job stress scale - Perceived general health	Prevalence of risky drinking was higher than the national average, particularly for younger (< 25 years) and mid-aged (45–54 years) workers. One in 6 CWs reported workmates being visibly affected by alcohol in the workplace. Key predictors of risky drinking were perception of alcohol-related risks to workplace safety, general health, alcohol knowledge and descriptive norms regarding workmates' alcohol use. Although job stress was positively correlated with AUDIT-C scores, it was not found to be a significant predictor of drinking in this study, indicating that stress was not a primary driver of alcohol in the sample.	8/8
Turner and Lingard (39)	Australia	To understand CWs work ability through exploring musculoskeletal bodily pain and the impact this has on construction workers' mental health.	Mixed methods approach	CWs (*n* = 67)	Phone interviews (10–50 min). - Musculoskeletal pain - Work ability - DASS-21	When broken down by age group, level of depression, anxiety, and stress were in the normal range for all age groups apart from participants in the 30–39 age group who experienced a mild level of anxiety. Participants whose pain had originated from work and who had upper neck and back pain, lower back pain, and pain in other joints had a significantly higher level of depression severity.	7/8
Zheng et al. (40)	China	To determine the relationship between occupational stressors and injury accidents.	Quantitative cross-sectional study	CWs (*n* = 105) and supervisors (*n* = 379)	- Scales by Cavanaugh - PANAS scale - Survey on occupational injuries - Questionnaire on task performance	Challenge stressors and hindrance stressors were positively related to occupational injuries, but only challenge stressors were positively related to attentiveness. Occupational injuries mediated the relationship between both challenge and hindrance stressors and task performance, while attentiveness mediated only the relationship between challenge stressors and task performance.	8/8
Alsulami et al. (5)	Saudi Arabia	To investigate the impact of emotional intelligence on workers' stress and safety behaviors.	Quantitative cross-sectional study	CWs (*n* = 265)	- Emotional intelligence - Stress	Emotional intelligence plays an important role to enhance the safety behaviors of the CWs besides reducing their workplace stresses. Furthermore, workers' stress levels are found to negatively impact their safety behaviors, indicating that any reduction in occupational stress can reciprocally enhance their safety compliance.	6/8
Dennerlein et al. (41)	USA	To identify work-related factors associated with the mental health and wellbeing of CWs	Mixed methods approach	−8 key informant interviews - 6 worker focus groups - 259 CWs	- JCQ. - Chronic work discrimination scale - Job precarity factors.	3 themes emerged from the interviews and focus groups: job demands and structure, social support and workplace relations, and job precarity. From the survey, higher psychological demands, higher work-to-family conflict, lower supervisor support, higher discrimination, and higher likelihood of losing a job were associated with higher psychological distress. When combined into a single model job, demands and work-to-family conflict remained significant.	8/8
Iremeka et al. (42)	Nigeria	To ascertain the effect of a group rational emotive behavior therapy (group REBT) on stress management among skilled construction workers in Nigeria.	Quantitative cross-sectional study	Skilled CWs (*n* = 160)	- PSS-14 - WIB-Q - Telegram group - Group REBT (8 weeks)	Results show that group REBT significantly improved stress and work-related irrational beliefs scores of the skilled construction workers after they were exposed to the intervention and compared with their colleagues in the control group. The significant reduction in stress and work-related irrational beliefs scores of the treatment group were also sustained at follow-up.	7/8
Liang et al. (43)	China	To reveal the influence of various coping behaviors on stress and safety among CWs.	Mixed methods approach	CWs (*n* = 314)	24 semi-structured individual interviews of 45 min.	Emotional stress in CWs can be positively predicted by confrontative coping, emotional discharge, and self-control, but can be negatively predicted by proactive coping. Similarly, physical stress is positively predicted by confrontative coping, self-control and avoidance, but negatively predicted by proactive coping. Non-compliance with safety rules was positively predicted by emotional stress, physical stress, and avoidance. No demographic factor was identified as a significant stress or safety factor for workers.	8/8
Choi et al. (44)	Korea	To determine the factors affecting job satisfaction during the disaster period by evaluating the job satisfaction of construction health and safety managers in special situations such as a pandemic, and to infer the overall job satisfaction and major factors based on the results.	Quantitative cross-sectional study	CWs (*n* = 227)	- General and work-related characteristics - The index of work satisfaction	The more working hours, the higher the working stress, so it was the highest when working more than 57 h. Job stress was significantly lower in the promotion opportunity variable, when they were unmarried and in charge of practical affairs (*p* < 0.05).	8/8
Frimpong et al. (45)	Ghana	To analyze the influence of age and work location on young workers' work-related mental health.	Mixed methods approach	Young CWs (*n* = 445)	Interviews, focus group discussion and quantitative survey instrument. - Work-related physical health - Work-related mental health	There was a high prevalence of the work-related substance abuse disorder, sleep problems, schizophrenia, and mania. No significant differences in the levels of work-related mental health problems were exhibited among different youth age sub-groups. Work location however accounted for significant differences in the levels of substance abuse disorder, sleep problems, anxiety disorder, and somatic symptoms exhibited.	6/8
Liang et al. (46)	China	To developed and test a model of the impact of COVID-19 pandemic perception on job stress of CWs.	Quantitative cross-sectional study	CWs (*n* = 498)	- Pandemic fear - Organizational pandemic response - Job stress - Coping behaviors - Job insecurity	Pandemic perception was significantly related to psychological and physical stress. Emotion-focused coping was mainly triggered by pandemic fear and job insecurity, while problem-focused coping was mainly triggered by organizational pandemic response. Furthermore, the effects of pandemic fear and organizational pandemic response on job stress were mediated by problem-focused coping.	6/8
Palaniappan et al. (47)	Singapore	To establish the prevalence of depression, anxiety and stress among foreign workers in the construction industry in Singapore.	Quantitative cross-sectional study	Foreign CWs (*n* = 348)	- DASS-21 - Work environment factors and conditions, shifts, leaves, accidents, etc.	About 29% of the study population exhibited moderate to extremely severe levels of depression; 37% showed moderate to extremely severe levels of anxiety; and 33% expressed moderate to extremely severe levels of stress. Ethnicity and lack of awareness of job scope were found to be significant predictors of all three parameters studied, namely, depression, anxiety and stress.	7/8
Palaniappan et al. (48)	Singapore	To determine the effectiveness of promoting peer support to reduce depression, anxiety and stress among migrant CWs in Singapore.	Quantitative cross-sectional study	Migrant CWs (*n* = 348)	Peer support training sessions. - DASS-21 (baseline and 6 months later). - Work conditions.	Statistically significant reduction was observed in measures of all the three parameters studied, namely, depression, anxiety and stress. A decrease of 3.3 (95% CI: 2.3–4.3) points in mean depression score, a decrease of 2.6 (95% CI: 1.6–3.7) points in mean anxiety score and a decrease of 2.7 (95% CI: 1.6–4.0) points in mean stress scores on the DASS-21 scale were recorded.	8/8
Segbenya and Yeboah (49)	Ghana	To explore the influence of occupational health and safety on CWs' performance in Ghana.	Quantitative cross-sectional study	CWs (*n* = 120)	- Attitudes toward occupational health and safety issues - Employee performance and the associated challenges	The construction sector lacks regular health and safety induction, orientation and refresher courses for CWs. Hence there are still occupational accidents and diseases affecting workers in the sector. For fear of being sacked, workers hardly report pains and injuries suffered at the construction sites.	6/8
Sushanthi et al. (50)	India	To analyze the relationship between depression, anxiety and CWs nicotine dependence according to their demographic and occupational characteristics in order to reduce the smoking related to stress which will help to develop indicators for smoking cessation strategies.	Quantitative cross-sectional study	CWs with the habit of tobacco (*n* = 416)	- GAD-7 - PHQ-9 - Fagerstrom test	16.6% of the participants had minimal anxiety, 28.4% of the workers had mild anxiety, 32.5% of workers had moderate anxiety, and 22.5% had severe anxiety. A positive correlation was found between nicotine dependence, GAD-7 [*r* = 0.82 and PHQ-9 (*r* = 0.79)].	8/8
Wu and Liu (51)	Taiwan	To incorporate formalism variables to explore their impact on the stress and anxiety of CWs during the epidemic.	Quantitative cross-sectional study	CWs in the leisure industry (*n* = 743)	Variables: policy formalism, COVID-19 fear, fear of infecting family members, fear of infecting self, anxiety, social support, and work stress.	COVID-19 fear positively affects anxiety and work stress; work stress mediates the relationship between COVID-19 fear and anxiety; fear of infecting family members and fear of infecting self both positively affect anxiety; policy formalism positively affects fear of infecting family members and fear of infecting self.	6/8

4DSQ, Four-Dimensional Symptom Questionnaire; AUDIT, Alcohol Use Disorder Identification Test; BCI, Brief Coping Orientation to Problems Experienced inventory (scale 0–3); CES-D, Center for Epidemiologic Studies Depression Scale; CWs, Construction Workers; DASS-21, Depression Anxiety Stress Scales (Depression: Normal 0–9/Mild 10–13/Moderate 14–20/Severe 21–27/Extremely severe ≥ 28//Anxiety: Normal 0–7/Mild 8–9/Moderate 10–14/Severe 15–19/Extremely severe ≥ 20//Stress: Normal 0–14/Mild 15–18/Moderate 19–25/Severe 26–33/Extremely severe ≥ 34); Distress Screener (scale 1–3: no = 0; sometimes = 1; or often = 2); FSSB, Family-Supportive Supervisor Behaviors; GAD-7, Generalized Anxiety Disorder scale; IES, Impact of Event Scale (scale 1–4, 0 = never, 1 = rarely, 3 = sometimes, 4 = often); FDW, Fatigue during work (scale 1–5/1 = 3 points; 2 = 2 points; 3 and 4 = 1 point; and 5 = 0 points); JCQ, Job Content Questionnaire; JSQ, Job Stress Questionnaire; K10, Kessler Psychological Distress Scale questionnaire; KOSS-SF, Korean Occupational Stress Scale Short Form; LMX, Leader, Member, Exchange; MBI, Maslach Burnout Inventory; MSD, Musculoskeletal Disorders; NMQ, Nordic Musculoskeletal Questionnaire; OSI, Occupational Stress Index (range: 65–75, scale 0–2; 0 = absent, 2 = highly present); PANAS, Positive and Negative Affect Schedule; PCQ, psychological capital questionnaire; PHQ-9, Patient Health Questionnaire; PPE, Personal Protective Equipment; PSS-14, The perceived stress scale-14; PTSD, depression and post-traumatic stress disorder; QEEW, Dutch Questionnaire on the Experience and Evaluation of Work [scale of 4 points (0 = never; 1 = sometimes; 2 = often; 3 = always)]; QoL, Quality of Life; REBT, Rational Emotive Behavior Therapy; SBS, Supervisor-Based Safety; PsyCap, Psychological capital; SF-12, 12 self-reported health questions; STAI, State-Trait Anxiety Inventory; TCI-RS, Temperament and Character Inventory Revised Short version; TEP, Team Effectiveness Process; TWH, Total Worker Health; WIB-Q, Work-related irrational beliefs questionnaire.

Of the 35 selected studies, 8 articles were conducted in China; 4 in the United States, Australia and Korea; 2 studies were conducted in Singapore, Ghana, and India; and 1 study was conducted in Canada, Ethiopia, the Netherlands, Indonesia, Nigeria, the United Kingdom, Pakistan, Saudi Arabia, and Taiwan. In 26 of the 35 selected articles, the sample consisted of construction workers in general, and in 3 of them a distinction was made between workers and supervisors. In another 2, the sample consisted of foreign or migrant construction workers, and the rest had specific characteristics, making a total aggregate sample of 13,399 subjects. As for the topic of research, 31 studies were found on stress, 10 on anxiety, and 3 on fear.

### Stress

The prevalence of substantial mental distress was between 16 and 50% among construction workers (20, 32) [20, 32]. A number of age-related stressors were identified (21, 30, 33, 39) [21, 30, 39, 33], as well as inappropriate safety equipment (18, 23) [23] and safety culture (5, 22–25, 31, 32, 34, 43) [5, 22–25, 31, 32, 34, 43], high workload (19, 41) [1, 41] and responsibilities (34), physical pain (20, 21, 27, 29), the psychological capital (35) and emotional intelligence (5), low participation in decision-making (19) and low social support from direct supervisor or co-workers (19, 23, 30, 33, 34, 41), the financial situation (26, 41), working hours (27, 28, 44), maladaptive coping strategies (28, 43, 46), the characteristics of the pandemic (46) and lack of knowledge (47). The results also revealed stress as a causative agent of occupational accidents (29, 36, 40).

### Anxiety

Between 37 and 50% of construction workers showed moderate to extremely severe levels of anxiety (47, 50). Among the risk factors to which construction workers may be exposed are those related to working conditions (25, 37, 45), working hours (28), substance use (28) and nicotine dependence (50), safety culture (37), age (39), high workload (37), lack of organizational justice and lack of reward (37), ethnicity and lack of knowledge (47), and the characteristics of the pandemic (51).

### Fear

Fear among construction workers was mainly associated with the characteristics of the COVID-19 pandemic (46, 51), with possible job insecurity (46), and with fear of losing their jobs (49).

## Discussion

The different studies showed multiple conditioning factors for stress, anxiety, and fear among construction workers such as age, inappropriate safety equipment, safety culture, high workload and long working hours, physical pain, low social support from direct supervisor or co-workers, lack of organizational justice and lack of reward, financial situation, maladaptive coping strategies, and characteristics of the pandemic.

The number of hours worked by employees is a determining factor for the level of stress according to the Occupational Stress Index (OSI). Several studies have found working hours of more than 12 h per day (27) or up to 47 h per week (28). This may lead to people not having enough time to spend with family/friends or to experimenting considerable fatigue, with the possible risk of injury or accidents of various kinds (22). This may require an understanding of the individual characteristics of workers in order to reduce the work-related stress generated by working hours and the associated lack of sleep that this may trigger (39).

In many cases, construction projects have to comply with a completion date, and must be finished within that timeframe with the resources that were initially planned. In this sense, many construction workers, despite being fatigued, continue to work for fear of losing their jobs, prioritizing the economic needs of their families over their physical health (27). Likewise, these long working days sharing space and tasks with other colleagues and superiors can be triggers of emotional stress related to an excessive mental workload (34) and at the physical level. The nature of construction work makes overexertion commonplace and routine, exposing the worker to frequent injuries that have a physical and mental impact on their daily life, both at work and in their social and family life (29). This could be explained by the job preservation mechanism, where people tend to work much harder when they perceive a threat of job loss (52). Similarly, financial strength can be a protective element or have a buffering effect on mental health problems in this area, as it allows individuals to meet their daily needs and have more resources to seek immediate mental health care (53).

On the other hand, several of the studies in this systematic review link the age of individuals to mental health (21, 30, 33, 39). In the work carried out by Yaldiz et al. (33), age was positively related to perceived stress. In contrast, the study by Turner and Lingard (39) found no relationship between stress, depression, and anxiety and age, but did find that only one age group, 30–39-year-olds, experienced a mild level of anxiety. Younger workers were more likely to be concerned about the amount and complexity of work than about their own ability, as they were inexperienced at this age at which they are likely to be unable to adequately cope with the additional workload. In addition, young workers more frequently overexerted themselves for significant periods of time and in the face of higher physical burdens (54). In contrast, a study in Ghana found no significant differences in levels of work-related mental health problems among different age subgroups of young people (45).

Safety culture is another factor that has been linked to higher levels of stress and anxiety (5, 22–25, 31, 32, 34, 43). In fact, many workers who are subjected to high levels of stress are more prone to accidents at work due to non-compliance with safety measures (43); i.e., the risk of accidents in stressed workers is up to 3.47 times more frequent than in unstressed workers (36). Similarly, masons tend to have little participation in decision-making, which, coupled with high work demand, low social support, and low organizational justice (55) may cause symptomatology consistent with stress, depression, and anxiety (19), thus increasing the risk of errors.

In this line, the low social support of the direct supervisor or co-workers is a key element as a protective or risk factor, depending on the case (19, 23, 30, 33, 34, 41). According to Bowers et al. (26), the most common stressors are lack of special events (86%), relationship problems with partners (68%), financial stress (62%), shift work (62%), and social isolation (60%). This phenomenon can lead many construction workers who believe they have mental health problems to be reluctant to participate in mental health programs or to seek help or support from family members, superiors, or medical services (39).

Finally, with appropriate coping techniques, construction workers can improve their stress levels. However, maladaptive coping techniques such as substance use (alcohol and drugs), self-distraction, denial, venting, among others, lead to increased depression, anxiety, and stress (28, 43, 46).

### Limitations

The present study has a number of limitations. Firstly, while the studies included in this review offered valuable contributions to knowledge about the mental health of construction workers, there are not enough studies that encompass the geographical dispersion and socio-cultural differences, types of construction work, and situations that can be encountered in the construction sector. This is why the results found in this review cannot be extended to all construction typologies, company types and sizes, and the important contextual variations that may exist in different regions of the world. Secondly, the multifactorial nature that can give rise to the different risk factors related to mental health makes it impossible to establish a precise interpretation of their cause, as many of these factors are found outside the workplace, such as education, culture, religion, family, or the personal condition of each worker, among others. In this sense, another limitation to be considered is the fact that the different working conditions established in each country at a global level and the laws and customs applied in each one of them in this area hinder the performance of a homogeneous analysis in general terms, as coping behaviors may differ considerably between one place or another, since certain working contexts that could a priori be considered susceptible to harming the health of the worker have become normalized.

## Conclusions

Accidents at work can be related to the mental health of workers, and age, hardship, and especially the long hours worked by construction professionals are factors that are significantly related to stress, anxiety, and fear. However, further studies are needed in this area that also include different work contexts and variables such as culture, education, professional qualifications, work environment, support systems, among others, in order to establish an early detection of risks.

The findings of this review could help construction companies to establish policies toward improving the working conditions of their employees and to increase knowledge about mental health in this sector. In this way, researchers and professionals dedicated to occupational safety, health, and risk prevention can identify these psychosocial factors and establish strategies and proposals to minimize the possible occurrence of such risk factors.

## Data availability statement

The original contributions presented in the study are included in the article/Supplementary material, further inquiries can be directed to the corresponding author.

## Author contributions

Conceptualization, formal analysis, investigation, writing—original draft, and writing—review and editing: CG-S, JG-I, JG-S, JF-R, JC-V, RA-C, JM-P, and CR-F. Data curation: CG-S, JC-V, CR-F, and JG-I. Methodology, resources, and visualization: CG-S, JG-I, JG-S, JF-R, JC-V, RA-C, and CR-F. Project administration: JG-S, JC-V, and CG-S. Software: CG-S, JG-I, and JG-S. Supervision: JG-S, JG-I, JF-R, RA-C, and CR-F. Validation: JG-I, JC-V, JG-S, RA-C, and JF-R. All authors contributed to the article and approved the submitted version.
