# Supplementary material for: Stress, fear, and anxiety among construction workers: a systematic review
Source: Front Public Health. 2023 Jul 13;11:1226914. doi: 10.3389/fpubh.2023.1226914 (PMC10372223; doi:10.3389/fpubh.2023.1226914)
Supplement: Supplementary file 1 [file Table_1.DOCX]

**SUPPLEMENTARY MATERIAL**

**Table S1. Scores of analytical cross-sectional studies**.

| **Studies** | **JBI** | **The participants and the environment are described in detail** | **Inclusion criteria are clearly defined** | **Exposure was validly and reliably measured** | **The criterion used to measure the condition was objective** | **Confounding factors were identified** | **Strategies for dealing with confounding factors** | **Results measured in a valid and reliable way** | **Appropriate statistical analysis was used** |
| --- | --- | --- | --- | --- | --- | --- | --- | --- | --- |
| (Leung et al., 2012) [18] | 8/8 | ☺ | ☺ | ☺ | ☺ | ☺ | ☺ | ☺ | ☺ |
| (Boschman et al., 2013) [19] | 6/8 | ☺ | ☺ | ☺ | ☺ | ☺ | ☹ | ☺ | ☹ |
| (Jacobsen et al., 2013) [20] | 8/8 | ☺ | ☺ | ☺ | ☺ | ☺ | ☺ | ☺ | ☺ |
| (Seo et al., 2015) [22] | 7/8 | ☺ | ☺ | ☺ | ☺ | ☺ | ☺ | ☹ | ☺ |
| (Leung, Liang, & Olomolaiye, 2016) [23] | 6/8 | ☺ | ☺ | ☺ | ☺ | ☹ | ☹ | ☺ | ☺ |
| (Chen et al., 2017) [24] | 7/8 | ☺ | ☺ | ☺ | ☺ | ☺ | ☹ | ☺ | ☺ |
| (Lim et al., 2017) [25] | 8/8 | ☺ | ☺ | ☺ | ☺ | ☺ | ☺ | ☺ | ☺ |
| (Bowers et al., 2018) [26] | 8/8 | ☺ | ☺ | ☺ | ☺ | ☺ | ☺ | ☺ | ☺ |
| (Chakraborty et al., 2018) [27] | 6/8 | ☺ | ☺ | ☺ | ☺ | 😐 | 😐 | ☺ | ☺ |
| (Langdon & Sawang, 2018) [28] | 8/8 | ☺ | ☺ | ☺ | ☺ | ☺ | ☺ | ☺ | ☺ |
| (Maqsoom et al., 2018) [30] | 7/8 | ☺ | ☺ | ☺ | ☺ | ☺ | ☺ | 😐 | ☺ |
| (Wang et al., 2018) [31] | 8/8 | ☺ | ☺ | ☺ | ☺ | ☺ | ☺ | ☺ | ☺ |
| (Widajati, 2018) [32] | 6/8 | ☺ | ☺ | ☺ | ☺ | 😐 | 😐 | ☺ | ☺ |
| (Yaldiz et al., 2018) [33] | 6/8 | ☺ | ☺ | ☺ | ☺ | ☹ | ☹ | ☺ | ☺ |
| (He et al., 2019) [35] | 8/8 | ☺ | ☺ | ☺ | ☺ | ☺ | ☺ | ☺ | ☺ |
| (Hussen et al., 2020) [36] | 6/8 | ☺ | ☺ | ☺ | ☺ | ☺ | 😐 | 😐 | ☺ |
| (Jung et al., 2020) [37] | 8/8 | ☺ | ☺ | ☺ | ☺ | ☺ | ☺ | ☺ | ☺ |
| (Roche et al., 2020) [38] | 8/8 | ☺ | ☺ | ☺ | ☺ | ☺ | ☺ | ☺ | ☺ |
| (Turner & Lingard, 2020) [39] | 7/8 | ☺ | ☺ | ☺ | ☺ | ☺ | ☹ | ☺ | ☺ |
| (Zheng et al., 2020) [40] | 8/8 | ☺ | ☺ | ☺ | ☺ | ☺ | ☺ | ☺ | ☺ |
| (Alsulami et al., 2021) [5] | 6/8 | ☺ | ☺ | ☺ | ☺ | ☹ | ☹ | ☺ | ☺ |
| (Dennerlein et al., 2021) [41] | 8/8 | ☺ | ☺ | ☺ | ☺ | ☺ | ☺ | ☺ | ☺ |
| (Iremeka et al., 2021) [42] | 7/8 | ☺ | ☺ | ☺ | ☺ | ☺ | ☹ | ☺ | ☺ |
| (Q. Liang et al., 2021) [43] | 8/8 | ☺ | ☺ | ☺ | ☺ | ☺ | ☺ | ☺ | ☺ |
| (Choi et al., 2022) [44] | 8/8 | ☺ | ☺ | ☺ | ☺ | ☺ | ☺ | ☺ | ☺ |
| (Frimpong et al., 2022) [45] | 6/8 | ☺ | ☺ | ☺ | ☺ | ☹ | ☹ | ☺ | ☺ |
| (H. Liang et al., 2022) [46] | 6/8 | ☺ | ☺ | ☺ | ☺ | ☹ | ☹ | ☺ | ☺ |
| (Palaniappan, Natarajan, et al., 2022) [47] | 6/8 | ☺ | ☺ | ☺ | ☺ | ☹ | ☹ | ☺ | ☺ |
| (Palaniappan, Rajaraman, et al., 2022) [48] | 8/8 | ☺ | ☺ | ☺ | ☺ | ☺ | ☺ | ☺ | ☺ |
| (Segbenya & Yeboah, 2022) [49] | 6/8 | ☺ | ☺ | ☺ | ☺ | ☹ | ☹ | ☺ | ☺ |
| (Sushanthi et al., 2022) [50] | 8/8 | ☺ | ☺ | ☺ | ☺ | ☺ | ☺ | ☺ | ☺ |
| (Wu & Liu, 2022) [51] | 6/8 | ☺ | ☺ | ☺ | ☺ | ☹ | ☹ | ☺ | ☺ |

Yes: ☺, No: ☹, Not clear or Not Applicable: 😐. JBI: Joanna Briggs Institute.

**Table S2. Scores of qualitative research**.

| **Studies** | **JBI** | **Congruence between stated philosophical perspective and research methodology** | **Congruence between research methodology and research question/objectives** | **Congruence between research methodology and methods used to collect data** | **Congruence between research methodology and representation and analysis of data** | **Congruence between research methodology and interpretation of results** | **Cultural and theoretical localisation** | **Influence of the researcher on the research and vice versa is addressed** | **Representation of participants and their voices** | **Ethical approval by an appropriate body** | **Relationship between findings and data analysis or data interpretation** |
| --- | --- | --- | --- | --- | --- | --- | --- | --- | --- | --- | --- |
| (Q. Liang et al., 2018) [29] | 8/10 | ☺ | ☺ | ☺ | ☺ | ☺ | ☺ | ☹ | ☹ | ☹ | ☺ |
| (Hampton et al., 2019) [34] | 9/10 | ☺ | ☺ | ☺ | ☺ | ☺ | ☺ | ☺ | ☺ | ☹ | ☺ |

Yes: ☺, No: ☹, Not clear or Not Applicable: 😐. JBI: Joanna Briggs Institute.

**Table S3. Scores of randomised controlled trials**.

|  | (Hammer et al., 2015) [21] |
| --- | --- |
| JBI score | 9/12 |
| True randomisation used for assignment of participants to treatment groups | ☺ |
| Allocation to treatment groups concealed | ☺ |
| Treatment groups similar at the baseline | ☺ |
| Participants blinded to treatment allocation | 😐 |
| Those delivering treatment blinded to treatment allocation | 😐 |
| Outcomes assessors blinded to treatment allocation | 😐 |
| Treatment groups treated identically other than the intervention of interest | ☺ |
| Follow-up complete and if not, were differences between groups in terms of their follow-up adequately described and analysed? | ☺ |
| Participants analysed in the groups to which they were randomised | ☺ |
| Outcomes measured in the same way for treatment groups | ☺ |
| Outcomes measured in a reliable way | ☺ |
| Appropriate statistical analysis used | ☺ |
| Trial design appropriate for the topic, and any deviations from the standard RCT design accounted | ☺ |

Yes: ☺, No: ☹, Not clear or Not Applicable. JBI: Joanna Briggs Institute
